# Case Report: Activating *PIK3CD* Mutation in Patients Presenting With Granulomatosis With Polyangiitis

**DOI:** 10.3389/fimmu.2021.670312

**Published:** 2021-04-28

**Authors:** Meiping Lu, Weizhong Gu, Yuanjian Sheng, Jingjing Wang, Xuefeng Xu

**Affiliations:** ^1^ Department of Rheumatology Immunology & Allergy, The Children’s Hospital, Zhejiang University School of Medicine, National Clinical Research Center for Child Health, Hangzhou, China; ^2^ Department of Pulmonary Medicine, The Children’s Hospital, Zhejiang University School of Medicine, National Clinical Research Center for Child Health, Hangzhou, China; ^3^ Department of Pathology, The Children’s Hospital, Zhejiang University School of Medicine, National Clinical Research Center for Child Health, Hangzhou, China; ^4^ Department of Nephrology, The Children’s Hospital, Zhejiang University School of Medicine, National Clinical Research Center for Child Health, Hangzhou, China

**Keywords:** activated phosphoinositide 3-kinase δ syndrome, immunodeficiency, granulomatosis with polyangiitis, *PIK3CD* gene, children

## Abstract

Activated phosphoinositide 3-kinase δ syndrome (APDS) is an autosomal dominant primary immunodeficiency caused by gain-of-function (GOF) mutations in *PIK3CD* or *PIK3R1* genes. The phenotypes of APDS are highly variable, ranging from asymptomatic adults to profound immunodeficiency causing early death in childhood. Herein, we reported two pediatric patients with APDS presented with recurrent lung infections, sinusitis, hematuria, and positive anti-neutrophil cytoplasmic antibody (ANCA), previously diagnosed as granulomatosis with polyangiitis (GPA). Bronchoscopy showed mucosal nodule lymphoid hyperplasia in the entire airway. Many inflammatory cells infiltrated around the airway and in the lung parenchyma, and numbers of CD3^+^ T cells and CD20^+^ B cells were significantly increased, especially CD3+ T cells. Whole exome sequencing showed that they had the E1021K (c.3061 G >A) mutation in the *PIK3CD* gene. These are the first reported cases of APDS presenting as childhood-onset GPA. Pediatricians should suspect of APDS in the differential diagnosis of children who present with GPA-like symptoms. Additionally, timely and repeated bronchoscopies could contribute to providing an important diagnostic clue for APDS.

## Introduction

Activated phosphoinositide 3-kinase (PI3K) δ syndrome (APDS) is an autosomal dominant primary immunodeficiency. It is caused by gain-of-function (GOF) mutations in PI3Kδ catalytic subunit p110δ (encoded by the *PIK3CD* gene) or regulatory subunit p85α (encoded by the *PIK3R1* gene) leading to APDS1 and APDS2, respectively (1, 2). The most frequent mutation found in APDS1 patients is E1021K (c.3061 G > A) in the *PIK3CD* gene. GOF mutations in the *PIK3CD* gene led to mTOR-mediated intrinsic CD8+ T cell defects, positioning p110δ as a critical player in regulation of human immunity. APDS is also termed PASLI disease (“p110δ-activating mutation causing senescent T cells, lymphadenopathy, and immunodeficiency”) (3). The phenotypes of APDS are highly variable, ranging from asymptomatic adults to profound immunodeficiency causing early death or necessitating HSCT in childhood (4). The children with APDS usually present with recurrent sinopulmonary infections, non-neoplastic lymphoproliferation, herpesvirus infections, autoinflammatory disease, and lymphoma (4). In general, pneumonia, bronchiectasis, and upper respiratory tract infections were common, often with childhood onset. Neurodevelopmental delay was also reported, suggesting a role for PI3Kδ in the central nervous system (4). Increased IgM levels, IgG deficiency, and CD4 lymphopenia were significant immunologic features (4). A study by Crank et al. also showed that patients with *PIK3CD* mutations presented with a classic hyper IgM phenotype during childhood and as young adults developed non-EBV-associated hematologic malignancies (5). Primary sclerosing cholangitis (PSC) not associated with *Cryptosporidium parvum* infection was also described in the adults (6). Patients with APDS may exhibit increased polymerized actin and increased apoptosis, likely leading to widespread necrotic skin lesions (7). Both mutations that reduce PI3Kδ activity and GOF mutations in PI3Kδ will cause defective lymphocyte development and function. Therefore, too little or too much PI3Kδ activity would lead to immunodeficiency (8).

Notably, recurrent sinopulmonary infections are a common manifestation for APDS, which is similar to granulomatosis with polyangiitis (GPA) presenting with upper airway, pulmonary, and renal involvement. Therefore, APDS patients only with recurrent sinopulmonary infections and renal involvement are likely to be diagnosed as GPA. Here, we report two pediatric patients with APDS presented with recurrent lung infections, sinusitis, hematuria, and positive anti-neutrophil cytoplasmic antibody (ANCA), previously diagnosed as GPA. The case report aims to make pediatricians pay more attention to the correlation between APDS and GPA.

## Case Presentations

### Patient 1

**Figure 1:**
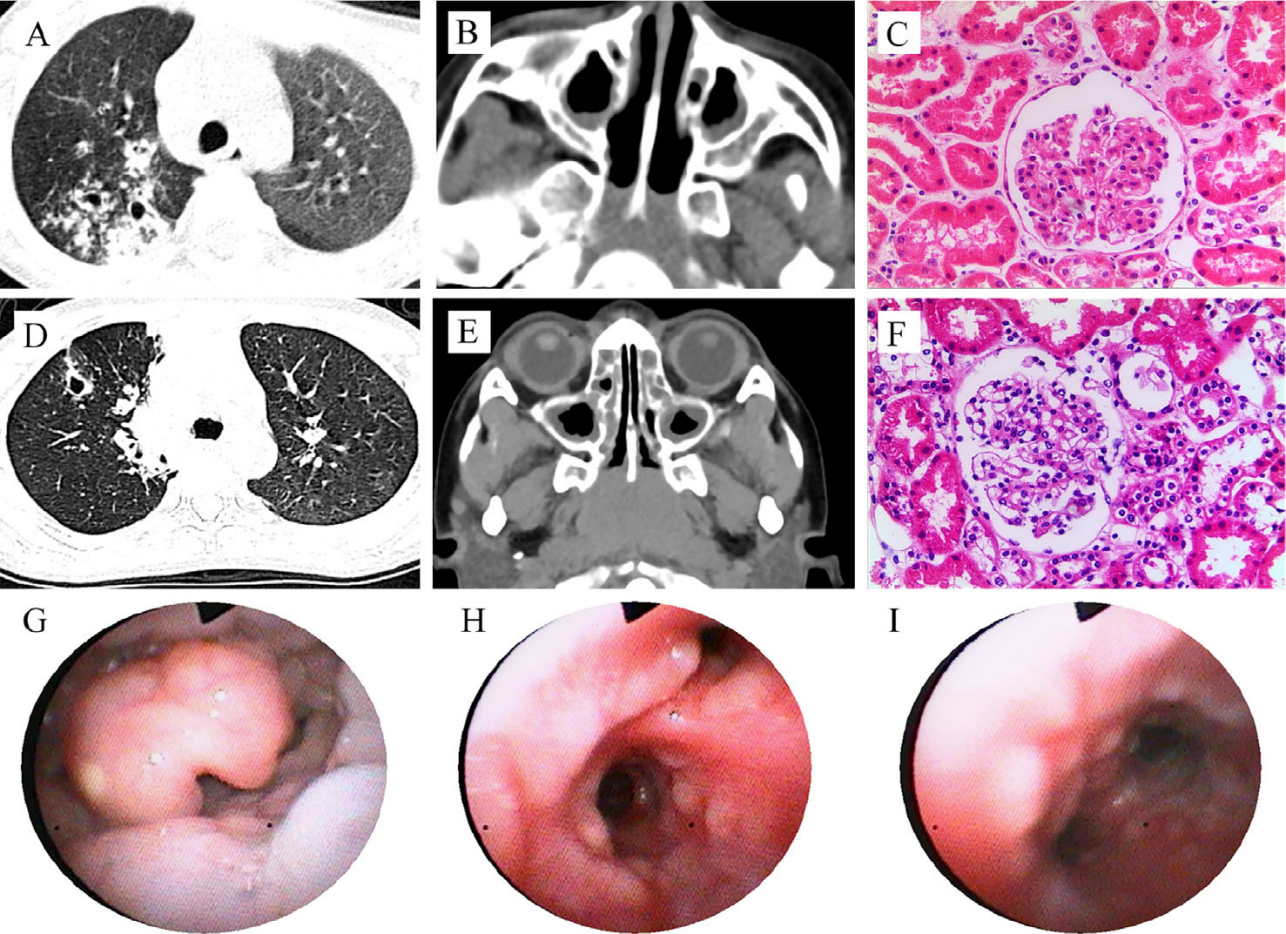
Clinical features of the patients with PIK3CD mutations. Patient 1 **(A–C)**: CT scan reveals pulmonary nodules with cavity formation, and patchy infiltration (**A**, right lung), and maxillary sinusitis **(B)**; Hematoxylin and eosin (HE) staining of renal biopsy showed no proliferation of capillary endothelial cells and mesangial cells in the glomeruli, no thickening of the basement membrane, and no glomerular sclerosis and interstitial fibrosis **(C)**. Patient 2 **(D–I)**: CT scan reveals pulmonary nodules with cavity formation, and patchy infiltration **(D)**, and maxillary sinusitis **(E)**; HE stains for renal biopsy showed minimal change, mild mesangial cell hyperplasia in glomerulus **(F)**, similar to patient 1; Bronchoscopy shows mucosal nodule lymphoid hyperplasia from pharynx to the entire airway **(G–I)**.

**Figure 2:**
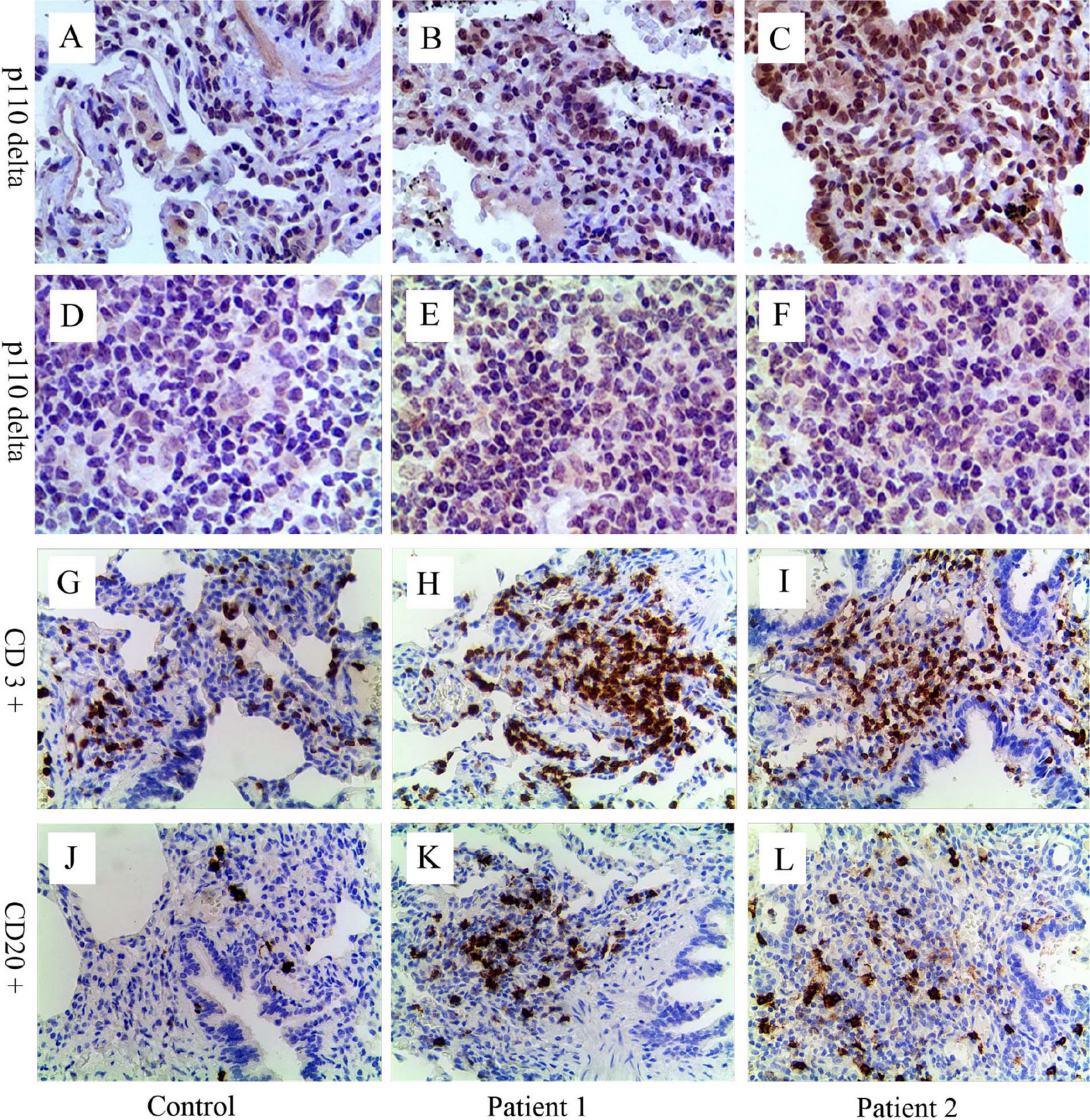
Immunohistochemical analyses for p110δ, CD3, and CD20. Compared with control **(A, D)**, expression of p110δ (brown signal) in lung tissue and lymph node sections from patient 1 **(B, E)** and 2 **(C, F)** significantly increased. Many inflammatory cells infiltrated around the airway and in the lung parenchyma **(H, I, K, L)**. Relative to control **(G, J)**, CD3^+^ T cells (**H**, **I**, brown signal) and CD20^+^ B cells (**K**, **L**, brown signal) significantly increased, especially CD3+ T cells. The control lung sample was from a child’s lung tumor resection, and lymph node sample was from a child with reactive lymph node hyperplasia.

In 2015, a 4-year and 6-month-old boy was admitted to our hospital for recurrent lung infections and hematuria for more than 2 years. Physical examination on admission revealed visible tonsils; cervical, axillary, and inguinal lymphadenectasis; dry rales in his both lungs; no murmurs in the heart; abdominal softness, no hepatosplenomegaly; no positive signs on nervous system. He had a BCG vaccination at birth with no adverse effects. Epstein-Barr (EB) virus IgM and cytomegalovirus (CMV) IgM were detected. High titers of anti-neutrophil cytoplasmic antibody (ANCA) for PR3 were detected (see **Table 1**). Chest CT showed pulmonary nodules with cavity formation, bronchiectasis, and patchy infiltration. Bilateral maxillary and ethmoid sinusitis were also seen in paranasal sinuses CT (**Figures 1A, B**). Ultrasound suggested the enlargement of liver, spleen, and superficial lymph nodes. Urine routine revealed increased urine red blood cells. Renal biopsy pathology indicated slight glomerular lesions (**Figure 1C**). Lymph node biopsy only demonstrated reactive hyperplasia. Bronchoscopy revealed no obvious abnormalities. Lung biopsy suggested an uneven distribution of lesions, and infiltrations of lymphocytes around the bronchiole with hyperplasia of collagen fibers (**Figure 2**). Bone marrow aspiration indicates hyperplasia of granulocytes.

**Table 1:** Clinical characteristics of the patients.

	Patient 1	Patient 2	References
Age	4y6m	7y9m	
Gender	Boy	Girl	
IgG (g/L)	20.8	19.35	6.36–14.04
IgG1 (g/L)	24.8	17.6	4.9–11.4
IgG2 (g/L)	1.87	0.47	1.5–6.4
IgG3 (g/L)	0.99	1.45	0.2–1.1
IgG4 (g/L)	0.01	<0.06	0.08–1.4
IgM (g/L)	2.49	1.98	0.29–1.21
IgA (g/L)	1.98	1.58	0.63–1.79
IgE (U/L)	4.3	18	<100
CD 20 (%)	4.91	23.96	14–21
CD3 (%)	73.46	63.57	64–72.5
CD4 (%)	32.06	25.58	29.5–35.5
CD8 (%)	37.58	31.78	24–33.5
CD3-CD16+CD56+ (%)	12.99	15.60	11–23
CD4/CD8	0.85	0.8	0.9–1.4
ANA	<1:80	1:80	<1:80
RF (U/ml)	255	26.1	0–14
ANCA (PR3)	+++	+++	–
Blood routine			
WBC counts (10^9^/L)	3.2	10.08	4–12
Hgb (g/L)	112	102	110–155
PLT (10^9^/L)	164	222	100–400
CRP (mg/L)	10	53	0–8
ESR (mm/h)		83	0–20
Urine routine			
RBC counts	1,637/µl	491/ul	0–13.6
WBC counts	10.3/µl	5.1/ul	0–13.2
Proteinuria	+	–	–
Proteinuria (mg/24 h)	106	189	<150
EBVEA-IgM	++	–	–
EBVCA-IgG	++	++	–
CMV IgM	+	+	–
BALF			
Macrophage (%)	59	64	>85
Lymphocytes (%)	11.0	15	≤15
Neutrophil (%)	30.0	21	<3
Eosinophil (%)	<1	<1	<1
RBC	+++	++	–

ANA, antinuclear antibody; ANCA, anti-neutrophil cytoplasmic antibody; BALF, bronchoalveolar lavage fluid; CMV, cytomegalovirus; CRP, C reactive protein; EBVEA, Epstein-Barr virus early antigen; EBVCA, Epstein-Barr virus capsid antigen; ESR, erythrocyte sedimentation rate; RBC, red blood cell; RF, rheumatoid factor; PLT, platelets; WBC, white blood cell. -, negative; + ∼ +++, positive. +++ refers to the most significant positive result.

The boy was diagnosed as GPA based on upper airway, lung, and kidney involvements, and positive ANCA (refer to EULAR/PRINTO/PRES criteria) (9). He was treated with oral prednisone at the initial dose of 1 mg/kg/day, tapered to 0.2 mg/kg/day by month 6, and 0.15 mg/kg/day as maintenance therapy. At the same time, he was given monthly intravenous cyclophosphamide infusion at the dose of 500mg/m^2^ for 6 months, and then mycophenolate mofetil was started as maintenance therapy. His hematuria improved. However, he still experienced recurrent lung infections within 2 years of follow-ups, and repeated bronchoscopy showed mucosal nodules. This patient was suspected of having lymphoproliferative disease and accepted genetic testing with the consent of his mother (his father not available). A mutation E1021K (c.3061 G >A) in the *PIK3CD* gene was found in this boy, while his mother had no mutation. He was then diagnosed with APDS1 and received regular immunoglobulin (400 mg/kg/month) and rapamycin treatment with the improvements of lung infection and hematuria. Considering his pulmonary infections, he was treated with piperacillin and Tazobactam, and then received Trimethoprim/Sulfamethoxazole prophylaxis for 6 months.

### Patient 2

Another patient was a 7-year and 9-month-old girl who presented with recurrent lung infections and hematuria for more than 6 years. During the past 6 years, she suffered from recurrent sinusitis, lung infections, and hematuria. The girl had positive ANCA and diagnosed as GPA. Although she received prednisone, her clinical manifestation failed to improve. Therefore, she was admitted to our Department of Rheumatology & Immunology in 2018. Physical examination revealed visible tonsils and cervical lymphadenectasis, but no clinical features suggestive of autoimmune disease. She had mild developmental retardation. CT also showed pulmonary nodules with cavity formation, and patchy infiltration, and bilateral maxillary sinus and ethmoid sinusitis (**Table 1**, **Figures 1D, E**). Abdominal ultrasound suggested hepatosplenomegaly and enlarged lymph nodes. Due to recurrent and refractory lung infections, she underwent bronchoscopy during hospitalization, showing mucosal nodule lymphoid hyperplasia in the entire airway (**Figures 1G–I**). Bone marrow and lymph node biopsies had no abnormal findings. Lung biopsy suggested inflammatory cells infiltration (**Figure 2**) and absence of granulomatous lesions, and renal biopsy pathology indicated mild glomerular disease (**Figure 1F**).

Due to mucosal nodule**s** of the airway, the patient was suspected of lymphoproliferative disease, and accepted genetic testing with the consent of her parents. Whole exome sequencing showed that she had a *de novo* mutation E1021K (c.3061 G >A) in the *PIK3CD* gene, while none of her parents had this mutation. In the end, she was diagnosed as APDS1, and received regular immunoglobulin (400 mg/kg/month) with reluctance to use rapamycin by her parents. In view of her pulmonary infections, she was treated with Cefoperazone and Sulbactam, and then received Trimethoprim/Sulfamethoxazole prophylaxis for 6 months. At present, her lung infections significantly improved.

Immunohistochemical staining of p110δ, CD3, and CD20 were performed in biopsy tissues of both patients taken previously (**Figure 2**). Increased expression of p110δ was found in histological sections of the lung and lymph node compared with the control, indicating increased recruitments of p110δ expressing cells (**Figures 2B, C, E, F**). Notably, significantly increased numbers of inflammatory cells were observed around airways and in the lung parenchyma, especially the infiltrations of CD3+ T cells (**Figures 2H, I, K, L**). This study was approved by the Ethic Review Board of Children’s Hospital, Zhejiang University School of Medicine (2019-IRB-105). Written informed consent was obtained from their legal guardians for the publication of any potentially identifiable images or data included in this article.

## Discussion

We described two pediatric patients with APDS1 who presented with GPA including recurrent lung infections, sinusitis, hematuria, and positive ANCA. They were treated successfully with regular immunoglobulin transfusion or immunosuppressive agent.

APDS1 is caused by a heterozygous GOF mutation in the *PIK3CD* gene encoding the p110δ catalytic subunit of PI3Kδ (2). The whole exome sequencing of the present two cases revealed the same E1021K (c.3061 G > A) mutation of the *PIK3CD* gene. The mutation was absent in both parents of patient 2, suggesting that it appeared *de novo.* The early stage of the two cases could mimic clinical characteristics of GPA, complicating the diagnose of APDS. Compared with GPA, children with APDS are more prone to suffering from CMV or EBV infections. Our cases also indicated that CMV and EBV were detected at the time of consultation, which might be correlated with immunesenescence and impaired cytotoxicity of CD8+ T and NK cells (10).

GPA is a systemic pauci-immune necrotizing small and medium-size vessel vasculitis associated with granulomatous inflammation, presenting with a triad of upper and lower respiratory tract involvements plus renal manifestation (11). ANCA can be detected in vasculitis, and anti-PR3 is more often seen in GPA (12). Additionally, GPA can also mimic pancreatic carcinoma, presenting with a pancreatic granulomatous necrotizing vasculitis in the lack of other visceral involvement and positive ANCA (13). Diversifications of clinical manifestations and negative ANCA would make the diagnosis of GPA difficult. However, our APDS1 children not only manifested classic symptoms of GPA, including recurrent lung infections, thick-walled cavitary lesions on chest CT, sinusitis, and renal involvement, but had positive ANCA (anti-PR3). Nichols-Vinueza et al. reported a 14-year girl with a *PIK3CD* mutation (c.1546 G > A), who had history of severe atopy, recurrent infections, elevated IgE and eosinophilia, and upper airway with nasal septal perforation and saddle nose deformity, but had no positive ANCA, previously diagnosed as GPA (14). So far, there is limited published literature on the association between ANCA and increased PI3Kδ activity. Augmented PI3Kδ signaling can lead to aberrant B cell development in bone marrow with increased proportions of immature B cells and reduction in mature recirculating B cells, contributing to impaired humoral immune response characteristic of patients with *PIK3CD* mutations (15, 16). In addition to abnormal B cell percentages (decreased in patient 1 and increased in patient 2), our cases showed a decreased CD4/CD8 ratio, associated with the production of autoantibodies such as ANCA. Patient 1 had positive ANCA in the early phase, and then ANCA detection was negative 2 years after diagnosis of APDS1. To date, patient 2 still has positive ANCA. She needs further follow-up to determine the ANCA titers. Nevertheless, whether ANCA titer alterations are consequence of therapeutic effect or early manifestation of disease development deserves further investigation. Notably, our cases further supported the fact that autoimmune conditions are an extremely common manifestation of APDS, accounting for about 30% patients (2). Among ANCA-associated vasculitis, GPA is the most frequent in Europe, while microscopic polyangiitis (MPA) was the most frequent in China (17, 18). Given the rarity of GPA in children, especially in China, GPA patients with poor therapeutic effects need further evaluation to rule out APDS.

Lymphadenopathy is a frequent manifestation of APDS. Some children also developed lymphoproliferation, autoimmunity, and lymphoma (19). Mucosal nodular lymphoid hyperplasia was visualized as cobblestone-like plaques or polyps in gastrointestinal tract and respiratory tract (4). In general, mucosal nodular lymphoid hyperplasia from respiratory tracts was rarely identified in our bronchoscopy practices. When mucosal nodule**s** of the airways were found in the present cases bronchoscopically, they were highly suspected of lymphoproliferative disease. Therefore, they accepted genetic testing, and subsequently mutations of the *PIK3CD* gene were found in both patients. Our case report also further revealed that mucosal nodular hyperplasia of respiratory tract was an important clue to the diagnosis of APDS. Moreover, some highly suspected patients could require repeated endoscopy to obtain the evidence of lymphoid hyperplasia.

A systemic review by Jamee et al. revealed that low IgG, IgA serum levels, and raised IgM levels were observed in most of APDS patients (2). Consistent with the features of APDS, the present patient 2 has low serum IgG2, and both patients have a mild elevation of IgM, possibly indicating a subtle class switching defect. However, our cases showed increased serum IgG levels, which could be associated with intravenous immunoglobulin replacement before hospitalization. Whether the increased IgG level is related to replacement therapy or caused by the APDS itself still need further investigation.

The treatments of APDS include conventional immunodeficiency therapy (immunoglobulin replacement), antibiotic prophylaxis, and hematopoietic stem cell transplant. Currently, the mTOR inhibitor, rapamycin, appears very attractive and useful in resolving lymphoproliferation (20). Our cases all received regular immunoglobulin, and only one child used rapamycin. Interestingly, their conditions improved significantly. Additionally, selective PI3Kδ inhibitors would have the potential to offer a targeted treatment option for APDS patients with greater efficacy and fewer side effects. However, administrations of the mTOR inhibitor and selective PI3Kδ inhibitor in children still need further follow-up and close observation.

## Conclusion

Due to the poor prognosis and the need to establish a prompt diagnosis and initiate appropriate treatment, pediatricians should suspect APDS in the differential diagnosis of children who present with GPA-like symptoms. Timely and repeated bronchoscopies may provide an important diagnostic clue of APDS, which should then be confirmed by genetic analysis.

## Data Availability Statement

The raw data supporting the conclusions of this article will be made available by the authors, without undue reservation.

## Ethics Statement

The studies involving human participants were reviewed and approved by the Ethic Review Board of Children’s Hospital, Zhejiang University School of Medicine (2019-IRB-105). Written informed consent to participate in this study was provided by the participants’ legal guardian/next of kin.

## Author Contributions

ML and JW collected and interpreted the data. WG and YS performed the experiments. XX and ML prepared the original draft of the manuscript. XX conceived and designed the study. All authors contributed to the article and approved the submitted version.

## Funding

This work was supported by fund from the National Natural Science Foundation of China (81871220). The funder had no role in study design, data collection and interpretation, or the decision to submit the work for publication.

## Conflict of Interest

The authors declare that the research was conducted in the absence of any commercial or financial relationships that could be construed as a potential conflict of interest.

## Abbreviations

ANCA, anti-neutrophil cytoplasmic antibody; APDS, activated phosphoinositide 3-kinase δ syndrome; GOF, gain-of-function; GPA, granulomatosis with polyangiitis; *PIK3CD*, phosphatidylinositol-4,5-bisphosphate 3-kinase catalytic subunit delta.
